# Cervicitis decidualis mimicking cervical cancer in pregnancy

**DOI:** 10.1016/j.gore.2024.101663

**Published:** 2024-12-16

**Authors:** Hooman Soleymani majd, Kezia Gaitskell, Catherine Johnson, Karin Hellner

**Affiliations:** a Oxford University Hospitals NHS Foundation Trust, Department of Gynaecological Oncology, Churchill Hospital, Oxford OX3 7LE, United Kingdom; b Oxford University Hospitals NHS Foundation Trust, Department of Pathology, John Radcliffe Hospital, Oxford OX3 9DU, United Kingdom; c Oxford University Hospitals NHS Foundation Trust, Department of Radiology, John Radcliffe Hospital, Oxford OX3 9DU, United Kingdom; d University of Oxford, Nuffield Department of Women’s & Reproductive Health, John Radcliffe Hospital, Women’s Center, Oxford OX3 9DU, United Kingdom

## Abstract

Cervical decidual reaction in pregnancy is a common finding. In rare cases, these changes can mimic the appearance of invasive cervical cancers.

We are presenting a case of a pregnant woman with a large cervical mass. Whilst magnetic resonance imaging (MRI) supported a diagnosis of cervical cancer, cervical tissue biopsies showed decidual reaction and no invasion. Cervical appearance postpartum reverted back to normal. Importantly, cervical biopsies were obtained in a fully equipped theatre attended by multiple specialities in case of life-threatening haemorrhage during pregnancy.

## Highlights

• Decidual reaction in pregnancy is a common finding.
• Rare ectopic deciduosis of the cervix can be mistaken for cervical cancer.
• Obtaining a biopsy in a controlled setting at an appropriate gestational age with a multidisciplinary set-up is critical.
• Cervical deciduosis spontaneously resolves postpartum.

## 1 Introduction

Decidualization refers to the morphological and functional changes that occur within the endometrium during pregnancy, to form the decidual lining into which the embryo implants. Ectopic decidualization is the presence of decidua outside the uterine cavity. Decidual reaction or deciduosis of the cervix is a benign growth produced by the hormone progesterone (Mittal et al., 2022). The true incidence is unknown but is estimated as high as 34% in pregnant women. Cervical deciduosis, also known as cervicitis decidualis, may look like cancer but is not malignant (Mittal et al., 2022, McGee and Slate, 1955, Nucci, 2002). Because it can cause complications during pregnancy, such as bleeding (Mangla et al., 2021, Oladipo and Mathew, 2005) and abnormal appearance it is often an incidental finding. The latter often results in cervical tissue sampling to exclude malignant causes.

## 2 Case presentation

A 28-year-old nulliparous woman presented to the local maternity assessment unit with unprovoked vaginal bleeding at 22 weeks gestation. She carried a singleton, low-risk pregnancy, and had no significant medical and surgical history. Her pregnancy had been uneventful until this point. Speculum examination revealed a friable, grossly abnormal looking cervix and urgent referral to colposcopy was initiated. She gave a history of normal cervical smears in the past.

**Fig. 1:**
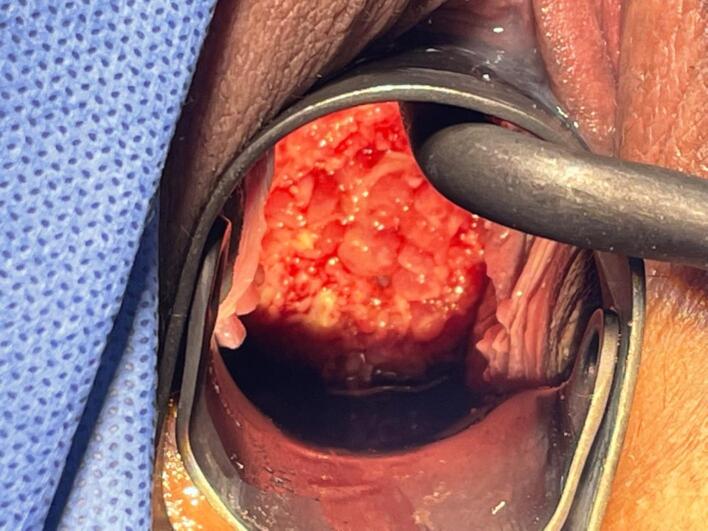
Intraoperative photograph of the polypoid decidual ectopy. The entire surface is replaced with cystic-polypoid lesions of varying size. Both epithelial tissue types, squamous and glandular, show oedematous changes. Upon application of acetic acid (not shown) the majority of surface epithelium exhibited dense acetowhite changes. There was increased surface vascularity and spiralling vessels seen.

Initial review in colposcopy was carried out jointly by two experienced colposcopists at 24 + 2 weeks gestation. Upon colposcopic visualisation (Fig. 1), the ectocervix showed an abnormal appearance, with hyperglandular protrusions and carpeted, cystic areas of varying size consuming the entire ectocervix. The abnormality expanded into the fornixes and upper vagina. Upon application of acetic acid, opaque acetowhite areas demarcated across the entire ectocervix. There were dense spiralling surface vessels present.

Gentle manipulation with a cotton bud – to expose the endocervical canal and vaginal fornixes during the examination – triggered brisk contact bleeding from the surface, which was difficult to control in outpatient colposcopy. The clinicians therefore refrained from obtaining biopsies in the outpatient department. On digital examination, a partially indurated and friable cervix was felt. A rectal exam was not performed at this point.

## 3 Investigations

**Fig. 2:**
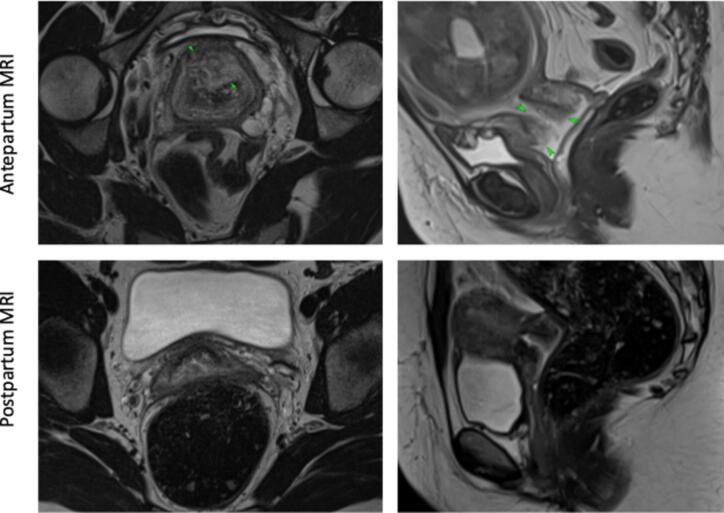
*Left panel*, the antenatal magnetic resonance image at 30 weeks gestation shows a large cervical mass in the centre (green arrow). *Right panel,* showing the same area 20 weeks postpartum with normal female genital anatomy and no cervical mass.

The findings raised the possibility of invasive cervical cancer and second opinion from the gynaecological oncology team was sought. A pelvic magnetic resonance imaging (MRI) was performed at 30 + 1/40 after the patient had missed earlier appointment invitations. The MRI (Fig. 2, top panel) showed a gravid uterus with a mass seen centred on the anterior cervix and extending into the vaginal fornix measuring 40 × 47 × 15 mm. There was no parametrial extensions, no pelvic lymphadenopathy and no metastatic lesions were seen in the pelvis. It was concluded that the mass was likely invasive cervical cancer, originating at the anterior cervix and expanding into the vaginal fornix. This gave a provisional FIGO stage of IIA2 cervical cancer.

In view of the suspected malignancy in pregnancy, the case was discussed at the departmental multidisciplinary team meeting. Following discussion at the multidisciplinary team meeting it was recommended to ascertain histopathological diagnosis of invasive disease by cervical biopsy.

The gynaecology team liased with the gynaecology oncology surgeons, obstretrics and neonatal teams and the decision was made to perform surgical tissue sampling in a fully equipped theatre under spinal anaesthesia after 30 weeks gestation, due to the significant perterm birth risk the procedure carries. The woman was admitted to the antenatal ward at 31 + 2 weeks gestation and consented for colposcopy, cervical biopsies and – in case of life-threatening bleeding – emergency caesarean hysterectomy.

**Fig. 3:**
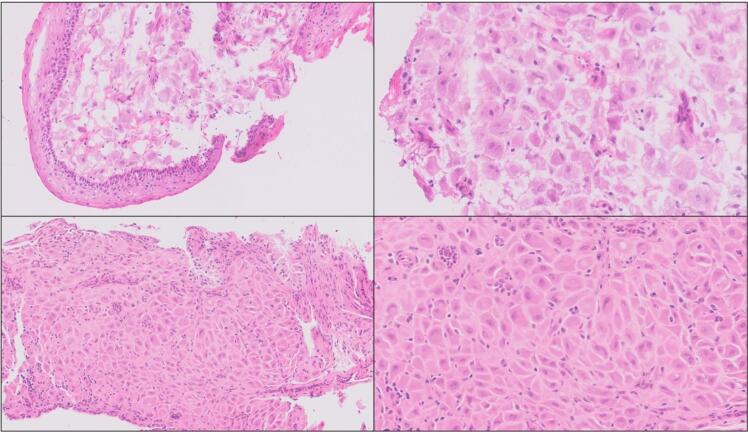
*Top panel,* Frozen section of cervical biopsy, showing squamous ectocervical epithelium with decidualisation of subepithelial tissues, with large polygonal cells with abundant eosinophilic cytoplasm. There is no significant nuclear atypia, and no mitoses are seen. H&E stain. Original image taken at ×20 magnification (left) and ×40 magnification (right). *Bottom panel,* Frozen-to-paraffin section of cervical biopsy. High-power image showing endocervical glandular epithelium with decidualised subepithelial tissues, with large polygonal cells with abundant eosinophilic cytoplasm. Significant nuclear atypia is absent, and no mitotic figures are seen. A few small clusters of acute inflammatory cells (neutrophils) are noted within the epithelium. H&E stain. Original image taken at ×20 magnification (left) and ×40 magnification (right).

Colposcopy in theatre showed that the abnormalities had progressed in size and cystic-polypoid appearance. Multiple cervical biopsies were obtained with a Tischler punch biopsy forceps. Brisk bleeding occurred immediately and was managed with diathermy to the area and haemostatic pressure. After no significant improvement, application of Floseal (a haemostatic matrix solution) and Surgicel (an absorbable cellulose-based haemostat) was used to achieve haemostasis. At the end of the procedure a vaginal pack was inserted for 24 hours. The woman was transferred to the delivery suite’s observation area and overnight monitoring of the mother and foetus was uneventful. The vaginal tamponade was removed on the next day, and the patient discharged without vaginal bleeding. Histopathology of the cervical biopsies (Fig. 3) showed florid decidual reaction with no evidence of malignancy. The finding was supported by immunohistochemistry staining showing weak and patchy estrogen receptor immunopositivity and negativity for p16, p53 and p40.

## 4 Outcome and follow-up

The woman remained under colposcopy surveillance until she delivered a healthy baby via caesarean section near term.

Postpartum colposcopy at three months showed gradual regression of the decidual changes. A cervical smear test taken three months postpartum tested negative for high-risk HPV and was not triaged for cytology in line with British cervical cancer screening guidance (NHS Cervical Screening Guidance, 2020). Cervical appearance six months postnatally was normal and cervical biopsies showed no evidence of an infection with high-risk human papillomavirus, cervical intraepithelial neoplasia or malignancy. Given the extent of the cervical tumour antepartum, a pelvic MRI was repeated 20 weeks postpartum (Fig. 2, bottom panel) showing normal pelvic anatomy and no cervical mass.

## 5 Discussion

Cervical deciduosis can be seen in one third of cervical biopsies from pregnant women. In rare cases, ectopic decidual reaction, such as disseminated peritoneal deciduosis can mimic metastatic disease (Cruz et al., 2014, Jadhav and Doshetty, 2022, Pigac and Masic, 2016, Pfaendler and Williams, 2022). Cytology can be misleading and can suggest high-grade dyskaryosis in the absence of any precancerous changes (Danos and Holmquist, 1967, Pisharodi and Jovanoska, 1995, Schneider and Barnes, 1981). Histopathology of decidualized cervical tissues show benign squamous mucosa with stromal decidual changes. Cervical decidualization spontaneously regresses after pregnancy within weeks of delivery.

Potential differential diagnoses include cervical polyps, cervical dysplasia, squamous cell or clear cell carcinoma of the cervix, metastatic cancers of other origin and Arias-Stella reaction – a hormone-related atypical change characterized by hypertrophy and vacuolization of glandular epithelial cells (Luks et al., 2012, Cove, 1979, Young and Scully, 1989).

As exemplary in our case, management involves multidisciplinary team decision making, often resulting in cervical tissue biopsy in second or third trimester in caesarean hysterectomy standby and close surveillance of the women by obstetrics and gynaecology units (Bolgarina et al., 2023, van Diepen et al., 2015). Timing of tissue sampling is crucial to avoid harm to mother and child, such as premature rupture of membranes and preterm labour and needs to be weighed out against potential delay of a cervical cancer diagnosis.

## 6 Authors’ contributions

KH and HS conceived the project. KH drafted the manuscript. HS, KG and CJ provided expert input and figures. KH and KG revised the draft. KH, HS, KG and CJ approved the final version of this manuscript.

## 7 Patient consent

The patient signed a consent form to authorize publication. This is available upon request.

## Funding source

No funding was sought to support this work.

## CRediT authorship contribution statement

**Hooman Soleymani Majd:** Writing – review & editing, Methodology, Investigation, Conceptualization. **Kezia Gaitskell:** Writing – review & editing, Methodology, Data curation. **Catherine Johnson:** Writing – review & editing, Methodology, Investigation, Data curation. **Karin Hellner:** Writing – review & editing, Writing – original draft, Project administration, Methodology, Investigation, Data curation, Conceptualization.

## Declaration of competing interest

The authors declare the following financial interests/personal relationships which may be considered as potential competing interests: HS, KG and CJ are employees of Oxford University Hospitals NHS Foundation Trust. KH is an employee of Oxford University. All authors declare no relevant conflict of interest.
